# Supplementary material for: A network dynamical simulation model for the study of antibiotic resistance in microbial communities
Source: Gut Microbes. 2026 Sep 19;18(1):2734703. doi: 10.1080/19490976.2026.2734703 (PMC13596919; doi:10.1080/19490976.2026.2734703)
Supplement: Supplementary file 1 [file KGMI_A_2734703_SM9053.zip › supplementary_file_1/Figures/ba_delta_gc_size_n100.pdf]

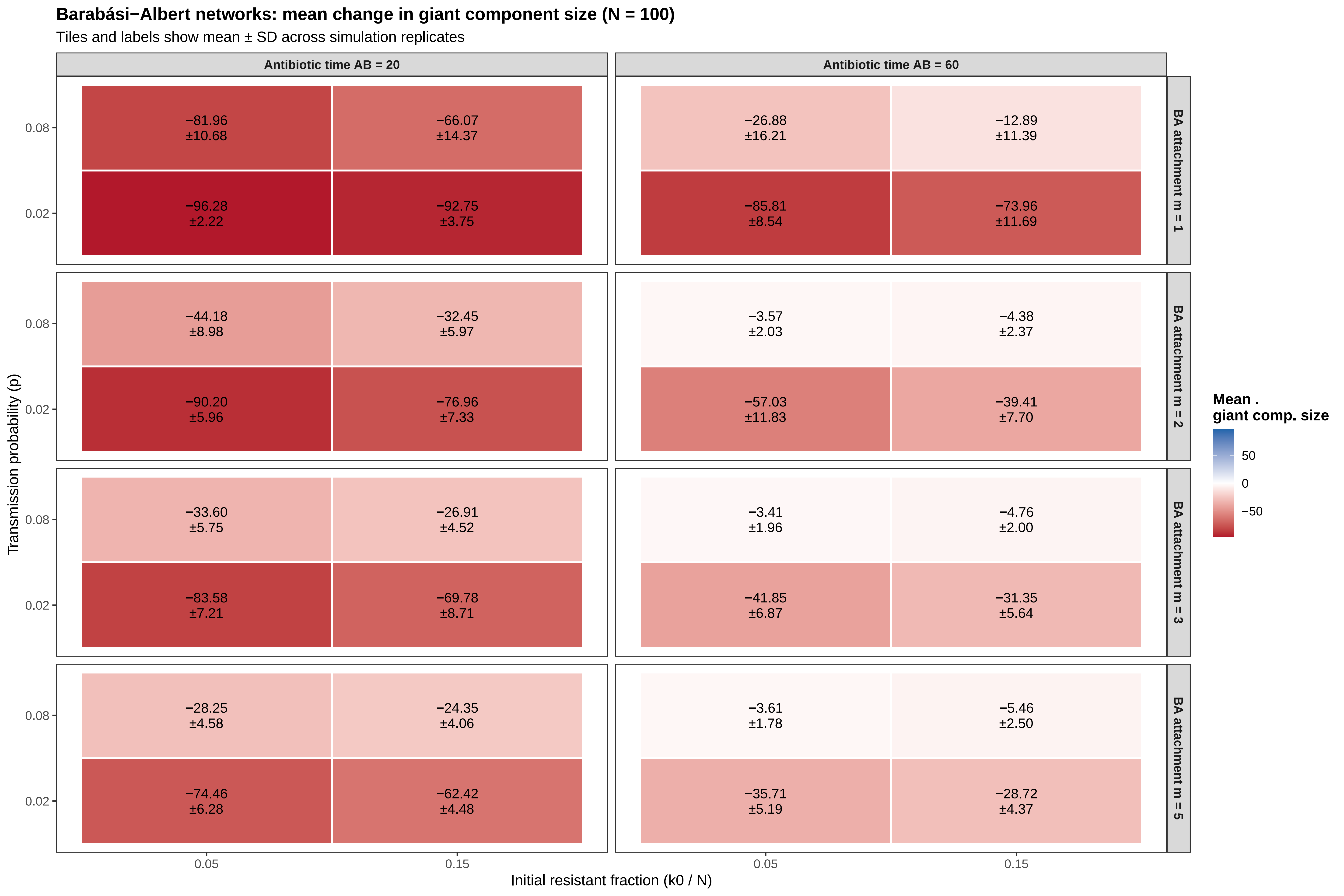
Barabási−Albert networks: mean change in giant component size (N = 100)
Tiles and labels show mean ± SD across simulation replicates
Antibiotic time AB = 20
Antibiotic time AB = 60
BA attachment m = 1
BA attachment m = 2
BA attachment m = 3
BA attachment m = 5
−81.96 ±10.68
−66.07 ±14.37
−96.28 ±2.22
−92.75 ±3.75
−26.88 ±16.21
−12.89 ±11.39
−85.81 ±8.54
−73.96 ±11.69
−44.18 ±8.98
−32.45 ±5.97
−90.20 ±5.96
−76.96 ±7.33
−3.57 ±2.03
−4.38 ±2.37
−57.03 ±11.83
−39.41 ±7.70
−33.60 ±5.75
−26.91 ±4.52
−83.58 ±7.21
−69.78 ±8.71
−3.41 ±1.96
−4.76 ±2.00
−41.85 ±6.87
−31.35 ±5.64
−28.25 ±4.58
−24.35 ±4.06
−74.46 ±6.28
−62.42 ±4.48
−3.61 ±1.78
−5.46 ±2.50
−35.71 ±5.19
−28.72 ±4.37
Transmission probability (p)
0.08
0.02
Initial resistant fraction (k0 / N)
0.05
0.15
Mean .
giant comp. size
50
0
−50
